# Evaluation of Mating Disruption for Suppression of *Plodia interpunctella* Populations in Retail Stores

**DOI:** 10.3390/insects16070691

**Published:** 2025-07-03

**Authors:** James F. Campbell, James Miller, James Petersen, Bill Lingren

**Affiliations:** 1 USDA, Agricultural Research Service, Center for Grain and Animal Health Research, Manhattan, KS 66502, USA; 2 Trécé Inc., Adair, OK 74330, USA; jmiller@trece.com (J.M.); jpetersen@trece.com (J.P.); blingren@trece.com (B.L.)

**Keywords:** mating disruption, stored products, post-harvest, pheromone, retail stores, integrated pest management, Indian meal moth, *Plodia interpunctella*

## Abstract

Mating disruption is a commercially available management tactic for pyralid moths, which are pests of stored products. However, evaluations of efficacy have had limited replication, which limits the ability to draw conclusions about its effectiveness or the impact of different variables on its efficacy. We evaluated the mating disruption of *Plodia interpunctella* in 33 retail pet supply stores (6415 to 17,384 m^3^) and the impact of factors such as insect density and application rate on efficacy. Prior to starting MD, the average capture of *P. interpunctella* was 40.2 ± 3.6 moths/trap/month. Immediately after starting treatment, there was a sharp drop in captures (67.8 ± 4.8%) and then a more gradual overall downward. Overall, under mating disruption, the average reduction was 85.0 ± 3.0%. Geographic location, initial moth density, and pheromone application rate did not significantly impact efficacy. Analysis of the relationships between moth captures and mating disruption dispenser density indicated that competitive mechanisms were the primary mechanisms involved. This was the largest replicated assessment of MD for the management of a post-harvest pest and provides valuable foundational and applied insights into the process. Our results show that a standardized MD program can provide pest suppression in retail stores, but it takes time to be fully effective. Finally, identifying the primary mechanism for efficacy provides important information needed for further refinement of MD programs.

## Simple Summary

Mating disruption is an insect pest management tactic that works by interfering with the ability of males to find females by releasing of large amounts of synthetic pheromones into the environment. Mating disruption is a commercially available tactic for pyralid moths, which are pests of stored products. However, evaluations of efficacy have limited replication, which limits the ability to draw conclusions about its effectiveness or the impact of different variables on its efficacy. Here, we evaluated the mating disruption of Indian meal moth in 33 retail pet supply stores (6415 to 17,384 m^3^) and the impact of factors such as insect density and application rate on efficacy. Immediately after starting treatment, there was a sharp drop in moth captures (68%) and then a more gradual overall downward trend over time. Geographic location, initial moth density, and pheromone application rate did not significantly impact efficacy. This was the largest replicated assessment of mating disruption for the management of a post-harvest pest, and it provides valuable foundational and applied insights into the process. Our results show that mating disruption can provide pest suppression in retail stores, but it takes time to be fully effective, likely because of immigration of mated individuals and inability to completely shut down mating in these complex environments.

## 1. Introduction

Mating disruption (MD) is a pest management tactic focused on the disruption of mate-finding behavior, typically based on the release of large amounts of synthetic sex pheromones into the environment. Mating disruption has a long history of research and commercial application as a pest management tactic for a variety of pest species, albeit predominately moths, and crop systems [1,2]. Pertaining to stored-product pest management, MD research has predominately been focused inside structures where food is processed and stored [3,4,5]. Most of the research and commercial development in post-harvest systems has focused on using (9Z,12E)-9,12-tetradecadien-1-yl acetate (ZETA), which is a pheromone component shared by multiple pyralid moth species, such as Indian meal moth, *Plodia interpunctella* (Hübner), tobacco moth, *Ephestia elutella* (Hübner), raisin moth, *Cadra figulilella* (Gregson), almond moth, *Cadra cautella* (Walker), and Mediterranean flour moth, *Ephestia kuehniella* (Zeller). Other stored-product pests that MD has been evaluated as a control method for include Angouimois grain moth, *Sitotroga cerealella* (Olivier) [6], cigarette beetle, *Lasioderma serricorne* (F.) [7], and European grain moth, *Nemapogon granellus* (L.) [8]. There are currently no commercial MD products available for these other species.

The evaluation of ZETA for the MD of stored-product pyralid moths started back in the 1970s, with early research evaluating efficacy in laboratory or simulated field experiments indicating that suppression of mating could be achieved [9,10,11,12,13,14,15,16,17]. More recently, research has focused more on evaluations of MD inside different types of food facilities using primarily experimental pheromone formulations, and varying levels of efficacy have been found [3,4,5,18,19,20,21,22,23,24,25]. Typically, these field tests have reported a reduction in moth captures in traps or a decrease in mated females or oviposition, but the size and duration of the reductions were variable, and complete shutdown of male captures or mating did not occur in most cases. A key limitation of much of this foundational research is the limited number of locations (i.e., replicates) evaluated for a particular treatment protocol. Furthermore, comparison of these studies is difficult due to the variation in experimental protocols, facility types, pest populations, and the use of different pheromone formulations. This variation limits the ability to generalize about efficacy or evaluate the impact of different factors like pheromone concentration, insect density, or geographic location on efficacy.

There are multiple competitive (e.g., competitive attraction, induced allopatry, and induced arrestment) and non-competitive mechanisms (e.g., desensitization, suppressed calling/mating, sensory imbalance, and camouflage) that can impact mating behavior [2]. MD can be a complex process, with many variables impacting efficacy, and multiple mechanisms of disruption may operate concurrently [2]. As a result, there are a limited number of studies that have clearly identified specific mechanisms, and the development of MD programs has largely been by trial and error. Miller et al. [26,27] and Miller and Gut [2] developed a theoretical basis for and graphical methods to evaluate the potential role of competitive and non-competitive mechanisms in MD, and they analyzed the available literature using this approach. Based on this strategy, we evaluated the potential mechanisms involved in *P. interpunctella* MD programs in retail stores using the data generated in this study and the methods provided by Miller et al. [26]. Understanding the behavioral mechanisms involved in MD can help lead to improvements in the implementation of MD programs.

Food in retail stores is vulnerable to infestation by stored-product insect pests, and *P. interpunctella* is one of the top pest species found in retail stores [28]. Pest management is challenging in the retail environment due to limitations on insecticides that are available, consumer concerns about insecticide applications, limited ability to shut down operations for treatment, inbound movement of infested food materials into the stores, climactic conditions that favor pest persistence, and tight margins in terms of money and labor for pest management all combined into an environment with structural features that make insect detection and targeting treatments difficult. In retail pet stores, there are unique challenges due to the presence of living animals, including birds, mammals, fish, and invertebrates, which further limit the use of insecticides. MD has many potential benefits in this type of environment, especially given the ability to work unobtrusively and safely when stores are open to customers.

Evaluating treatment efficacy at the scale and under the conditions necessary to be applicable and generalizable to end-users is often beyond the scope of an individual scientist’s research program. To answer these larger-scale questions, it is necessary to develop collaborative programs and rely on industry collaborators to help with the data collection. The research reported here is an example of collaborative research involving different segments of the food manufacturing, retail, and pest management industries. The original project objectives were developed by Nestle Purina Petcare in collaboration with two retail pet store chains but were expanded over time to include Trécé Inc. (the company that manufactures the MD product) and USDA ARS. This group provided the locations, labor, and materials needed to evaluate the effectiveness of MD with a relatively high level of replication. With this collaboration, we tried to balance the need to develop a simplified program that can be readily applied to commercial systems and the need to rigorously evaluate MD program efficacy.

The objective of the project reported here was to evaluate how well MD can suppress moth populations in retail pet stores by assessing treatments under ‘real-world’ conditions found in commercial operations. Through collaborations, we were able to have much higher replication than previous MD evaluations, allowing us to also test some important factors that need to be considered, including pheromone dispenser density, spatial distribution of pheromone dispensers, and pest population density. The MD program evaluated in this study was designed to be simplified and standardized so that it could be applied across multiple locations as part of a commercial pest management contract, which required an allowance for flexibility in the methodology based on industry needs.

## 2. Materials and Methods

This study was conducted at 33 commercial retail pet supply stores from two different store chains. The average volume for the stores was 10,834 ± 527 m^3^, with stores ranging between 6415 and 17,384 m^3^ (Table 1). The average volume of stores in chain #1 was 13,013 ± 674 m^3^, which was slightly larger than for chain #2 at 8785 ± 367 m^3^. All the stores were located in the contiguous United States of America, and based on the United States National Climatic Data Center classification of climate regions, there were 6 stores in the southwest, 6 stores in the south, 15 stores in the southeast, 6 stores in the combined central (Ohio Valley) and northeast regions (Table 1). The central and northeast regions, which each contained 3 stores, were combined for analysis because of the small number of stores in these two regions and the similarity in climatic conditions. Although the environmental conditions inside the stores were controlled and similar across geographic regions, location could influence seasonal patterns of outside insect activity and resulting immigration pressure and patterns in inbound product infestation issues, given that stores within the same retail chain and geographic region are more likely to be supplied by the same distribution centers.

The single CIDETRAK^®^ IMM and MESO (Trécé, Adair, OK, USA) were the mating disruption formulations used in this study. The single dispensers contained 160 mgs of the active ingredient, whereas the MESO dispensers had 640 mgs of active ingredient and were the equivalent of four single dispensers. There were three phases to this study, and different dispenser types, mounting locations, and dispenser densities were used during each phase. Pheromone dispensers were changed between study phases or according to the manufacturer’s recommended interval of 130 to 150 days.

The original goal of the project was to determine how well mating disruption could work in a retail store environment and if a standardized, simple, and easy-to-apply application method could be effective across stores. In phase 1, an initial evaluation of single dispensers was conducted at 7 stores. During phase 1, single CIDETRAK^®^ IMM dispensers were used, and the number per store varied, as well as whether the dispensers were mounted on shelves or from the ceiling (Table 1). Phase 1 lasted 8-16 months, depending on the store. These 7 stores continued to be used in phases 2 and 3. In phase 2, 9 MESO dispensers per store were used instead of single dispensers in 27 stores (Table 1). The goal of this change was to reduce the labor involved in placing the dispensers within the store. Labor was reduced by using fewer dispenser locations per store and mounting them on shelves rather than the ceiling. However, initial observations of the results under phase 1 and phase 2 suggested that the program was not working as consistently as desired. It was determined that in phase 2, some stores ended up with an application rate below the label (Table 1). Phase 2 lasted 5 to 6 months, depending on the store.

Under phase 3, three different dispenser densities were used per store. Unlike phase 2, only single dispensers, rather than MESOs, were used. The dispensers were spaced as evenly as possible through the store, and rather than hanging them directly on the shelves, they were attached to a piece of metal wire that was held to the shelving units with magnets (dispenser suspended ~66 cm above the top of the shelf). This method of mounting was predicted to provide better dispersal of the pheromone and reduce labor involved in setting up the program.

During phase 3, an additional 6 stores were added that used MESO dispensers, but they were deployed at a higher rate and with more mounting locations per store than was used under phase 2. The number of dispensers ranged between 42 and 84 single dispenser equivalencies, with an average of 59 ± 8 single dispenser equivalence per store.

Throughout phase 3, the three rates used corresponded with the low, medium, and high rates on the label. Because of the desire to use a standard method across stores, set numbers of dispensers were used per store, but because of variation in store size, there was variation in the volume treated per dispenser. The three rates were 36, 54, and 72 dispensers per store. The average volume treated per dispenser was 302.3 ± 29.7 m^3^ at the 36-dispenser rate (*n* = 10), 209.7 ± 18.3 m^3^ at the 54-dispenser rate (*n* = 8), and 136.1 ± 10.1 m^3^ at the 72-dispenser rate (*n* = 9). Stores were selected for a specific application rate so that the rates were as evenly distributed between the two retail chains and geographic areas and across a range of initial moth capture levels as possible. The additional 6 stores added in phase 3, treated with MESO dispensers, had an average of 190.9 ± 0.3 m^3^ treated volume per dispenser.

Moth activity in the stores was monitored using eight pheromone-baited diamond sticky traps (STORGARD^®^ II traps with IMM+4 pheromone lures, Trécé, Adair, OK, USA) hung on shelves. In the 6 MESO-treated stores of phase 3, a greater number of traps (20–25) was used. Traps were as evenly spaced as possible, and the stores typically had at least one trap located in dry cat food, dry dog food, wild bird seed, cat litter (because some types are grain-based), and the receiving room (which has a loading dock which opens to the outside and shelving to store inbound and outbound products). Traps were checked and replaced monthly, and the total number of *P. interpunctella* moths captured per trap was counted. Trapping data is presented as the mean ± SEM of moth captures/trap/month in each store. A pre-MD monitoring period (3.6 ± 0.3 months/store, Table 1) was included for each store to provide the control population level for that store. Separate control stores were not monitored because of practical constraints with cooperators not wanting to contribute untreated retail stores and concerns at the start of the project about the level of variation in populations among the individual stores.

Levels of moth capture were compared using *t*-tests (normal distribution) or Mann–Whitney’s U tests (non-normal distribution) for paired comparisons. Analysis of variance (ANOVA) was used for multiple comparisons and for determining if there were linear relationships in the data when the data was normally distributed. The Kruskal–Wallis analysis of variance on ranks was used when the data was not normally distributed. Analytical methods described and applied in Miller et al. [26,27] and Miller and Gut [2] were used to assess the MD behavioral mechanism involved. This is a graphical approach with competitive and non-competitive mechanisms generating different relationships in plots of number of dispensers (x) with untransformed moth captures (y), number of dispensers (x) with 1/moth captures (y), and number of dispensers * moth captures (x) with moth captures (y). For these graphical assessments, we used the moth captures prior to starting MD for the control zero dispenser data point on the x-axis, since separate control stores were not included in this study.

## 3. Results

### 3.1. Analysis of Combined Stores

**Figure 1:**
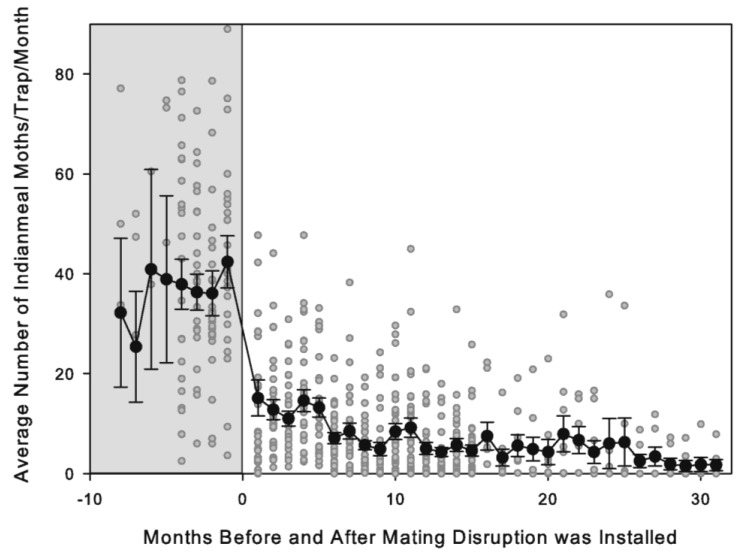
Number of *Plodia interpunctella* males caught in pheromone-baited traps per one-month monitoring period before and during mating disruption (MD) treatment in retail pet stores (black circles: mean ± SEM of all the stores; gray dots: individual store means; grey section indicates prior to start of MD treatment.

Prior to starting the MD program, the average capture rate of *P. interpunctella* was 40.2 ± 3.6 moths/trap/month. Levels of moth capture prior to starting MD program did not differ among the different geographic regions (ANOVA: *F* = 0.933; d.f. = 3, 32; *p* = 0.437): the number of moths/trap/month was 34.7 ± 11.3 in the combined central and northeast region, 34.0 ± 10.0 in the south region, 46.7 ± 4.0 in the southeast region, and 35.7 ± 8.2 in the southwest region. The two retail store chains also did not differ in their initial pre-MD moth captures (46.7 ± 5.6 compared to 34.1 ± 4.1 (*t*-test: *t* = 1.848, *p* = 0.074)). While moth captures varied over time and among stores prior to start of MD, they typically remained high and did not exhibit any seasonal trends (Figure 1).

During MD (combined data from all phases), the average number of *P. interpunctella* captured was 4.6 ± 0.7 moths/trap/month, which represented a reduction of 85.0 ± 3.0%. In the last monitoring period of the study, the average number captured was 3.9 ± 1.0 moths/trap/month. There was no difference in moth captures under MD among the geographic regions (ANOVA: F = 0.427; d.f. = 3, 32; *p* = 0.735): the mean ± SEM of the number of moths/trap/month was 4.7 ± 2.2 in the combined central and northeast region, 3.5 ± 1.9 in the south region, 5.3 ± 0.8 in the southeast region, and 3.7 ± 1.4 in the southwest region. The two retail store chains also did not differ in moth captures under MD (4.8 ± 1.1 compared to 4.3 ± 0.8 (Mann–Whitney test: *U* = 274.5, *p* = 0.942)). Given the lack of differences between geographic region and store chain, only combined data was used for subsequent analysis.

In phase 1 of the study with the seven stores, the reduction in captures under MD was 64.4 ± 15.2%, with an average capture rate of 11.9 ± 2.6 moths/trap/month (one store had an increase after MD installation). During phase 2 of the study, in the 27 stores with nine MESO MD dispensers, the reduction in captures from the levels prior to starting MD was 72.6 ± 3.8%, with an average capture rate of 6.9 ± 1.4 moths/trap/month. For just the stores that did not have any MD treatment prior to MESO installation (*n* = 20), there was a 73.1 ± 3.1% reduction and average capture rate of 6.1 ± 1.6 moths/trap/month, which is similar to the levels in the stores that had a previous MD treatment.

Immediately after starting MD in the stores, there was a sharp drop in moth captures and then a more gradual overall downward trend in captures over time (Figure 1). Combining all the different trials, the percentage reduction in the first monitoring period after starting MD was 67.8 ± 4.8%. For just the seven stores starting in phase 1 with single dispensers, the immediate reduction was 63.3 ± 12.1%, for the twenty stores starting in phase 2 with nine MESOs, the immediate reduction was 63.1 ± 6.4%, and for the six stores starting in phase 3 with MESOs, the immediate reduction was 77.9 ± 7.5%. In the last sampling period of the study, the average capture rate was 3.9 ± 1.0 moths/trap/month, and this represented an 88.4 ± 2.9% reduction from the pre-MD average.

### 3.2. Individual Store Analysis

**Figure 2:**
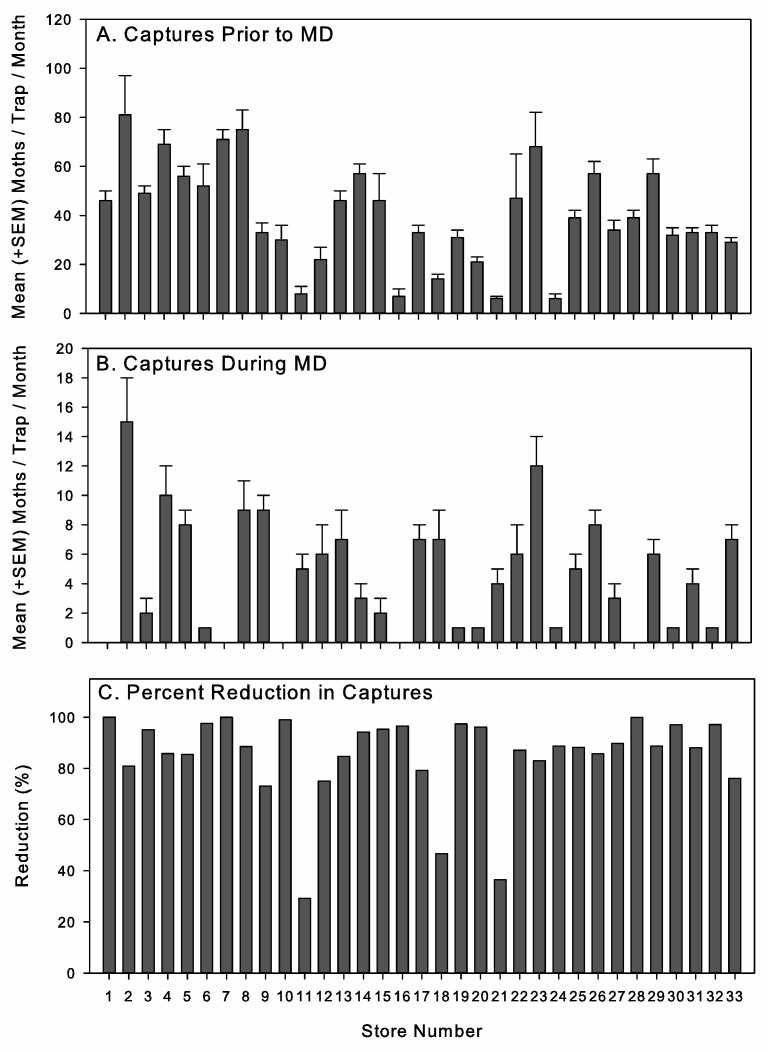
*Plodia interpunctella* captures in pheromone-baited traps per one-month monitoring period (**A**) prior to starting mating disruption (MD) and (**B**) during MD (mean + SEM), and (**C**) percentage reduction in captures under MD for each individual retail pet store included in the study.

Although the stores shared many common features, there was considerable variation in the number of *P. interpunctella* captured prior to starting the MD program (Figure 2A) and during the MD program (Figure 2B) among individual stores. This probably reflects differences among the stores in their pest management, stock rotation, and sanitation practices, although it was not possible to quantify this fully given the limited visitations to the stores. The percentage reduction in moth captures under MD was relatively high across the stores, with the few exceptions tending to be stores that had a lower moth density prior to the start of the program (Figure 2C).

### 3.3. Insect Abundance and Location Within Store

**Figure 3:**
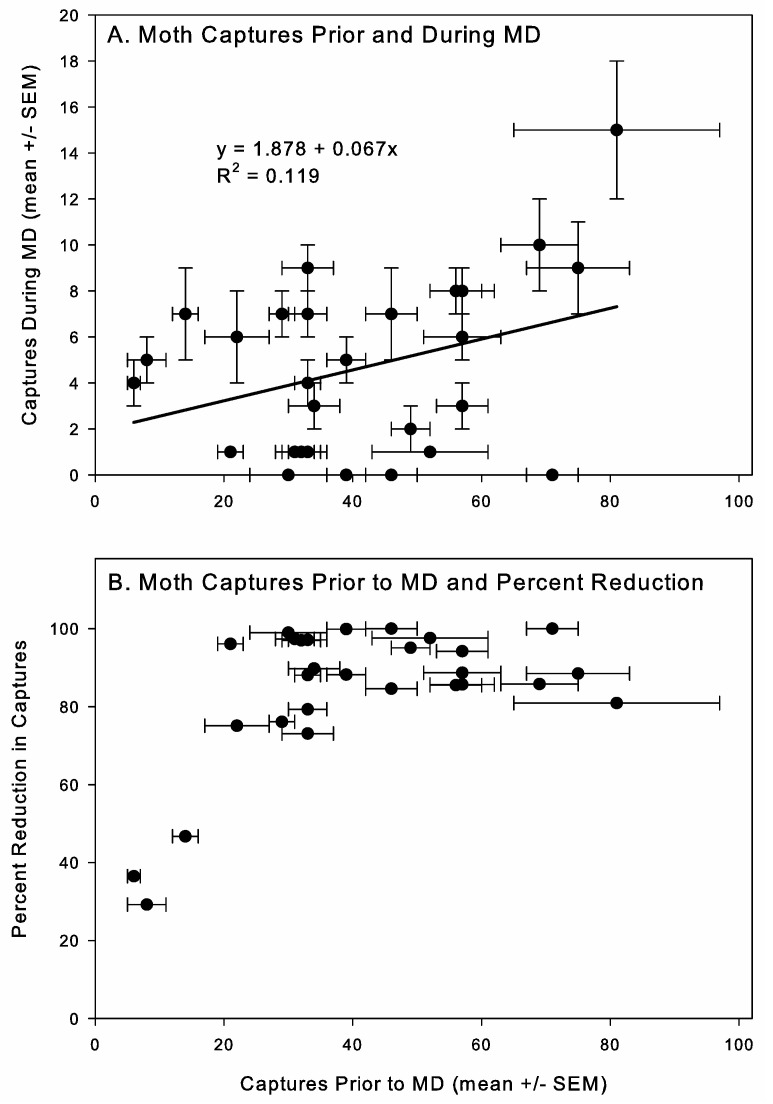
Correlation between *Plodia interpunctella* captures in pheromone-baited traps per one-month monitoring period (mean ± SEM) in retail pet stores prior to starting mating disruption (MD) and during MD treatment (**A**), and relationship between captures prior to starting MD (mean ± SEM) and percentage reduction in captures during MD (**B**).

MD is predicted to be less effective at higher insect densities when competitive mechanisms are involved, but there was not a significant relationship between average number of moths captured per store prior to MD and the number of insects captured under MD (F = 3.502; d.f. = 1, 26; *p* = 0.073). There was a slight upward trend to the data, but the R^2^ value was relatively low (Figure 3A). Plotting the relationship between the percentage reduction and moth capture levels prior to MD, it was consistently between a 70 and 100% reduction (Figure 3B), except in the stores with very low initial moth densities, where reduced efficacy is likely an artifact due to variation in captures observed over time.

**Figure 4:**
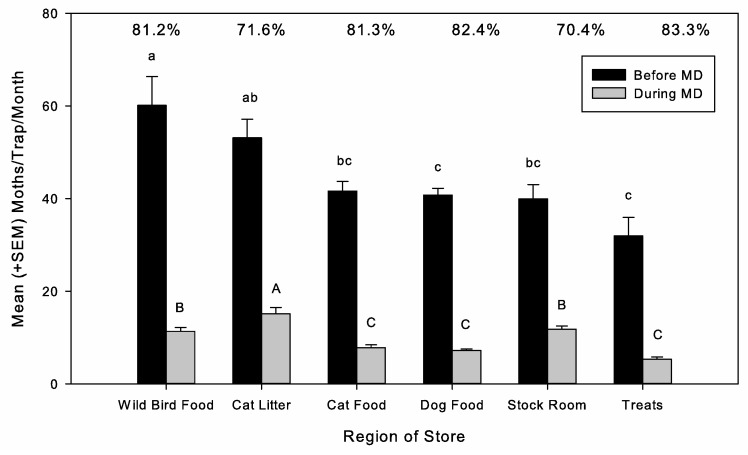
*Plodia interpunctella* captures in pheromone-baited traps per one-month monitoring period (mean + SEM) in retail pet stores prior to starting mating disruption (MD) (black bars) and during MD treatment (gray bars) for different regions in the stores; bars of the same color with the same letters did not have significantly different means (*p* < 0.05), and the percentages shown at the top of the graph represent the percentage reduction in moth captures between before and during MD.

Traps placed in different sections of the stores varied in terms of moth captures prior to starting MD (ANOVA: F = 7.99; d.f. = 5, 857; *p* < 0.0001), with the highest levels of capture observed in wild bird seed and cat litter sections (Figure 4). The percentage reduction in moth captures under MD was similar among store sections, ranging between 70.4 and 83.3%. During MD, average moth captures were much lower in each section, but there were differences among the sections (ANOVA: F = 23.51; d.f. = 5, 3744; *p* < 0.0001). Under MD, moth captures were highest in cat litter sections, then next highest in the receiving room and wild bird food sections, and the lowest captures were in cat food, dog food, and treats sections of the stores (Figure 4). These were also the areas where signs of moth infestation were most frequently observed and may reflect areas most vulnerable to infested inbound product. The high activity in the cat litter section was surprising, but infestation was found to be associated with cat litter made with grain products, and *P. interpunctella* can develop on these materials. The high captures in the receiving room were probably due to damaged and infested material being stored there prior to return or disposal but could also have been due to openings to the outside environment.

### 3.4. Dispenser Number and Efficacy

**Figure 5:**
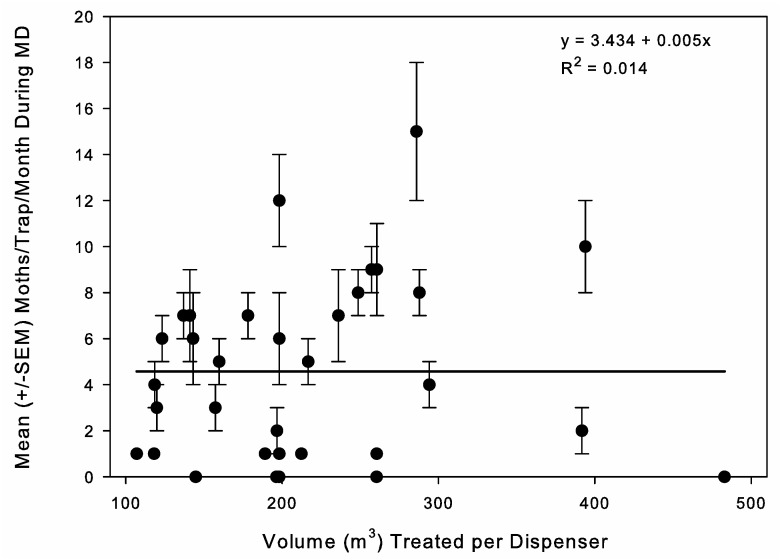
Correlation between *Plodia interpunctella* captures in pheromone-baited traps per one-month monitoring period (mean ± SEM) in retail pet stores during mating disruption (MD) and the volume of space treated per MD dispenser.

**Figure 6:**
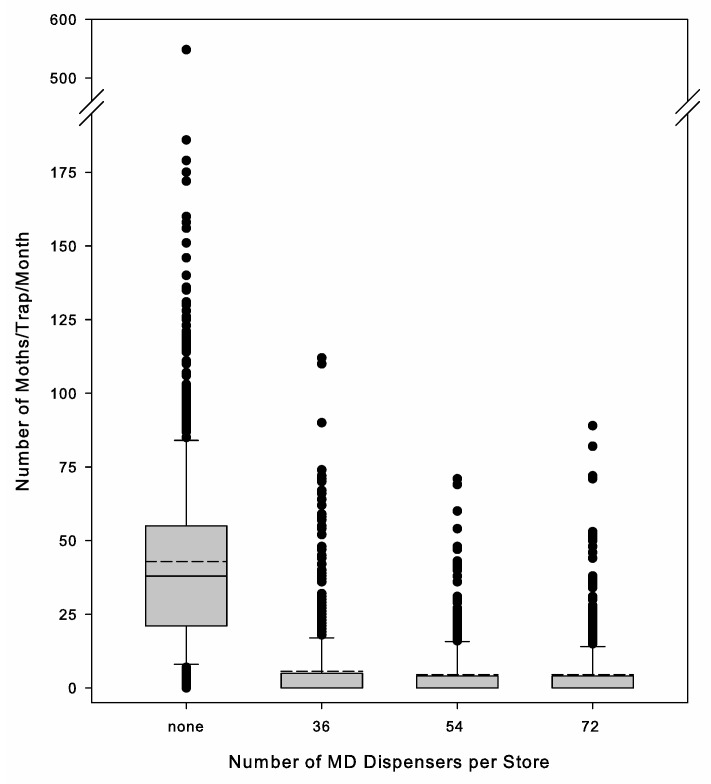
Box plots of *Plodia interpunctella* captures in pheromone-baited traps per one-month monitoring period prior to starting MD (none) and with 36, 54, and 72 mating disruption (MD) dispensers (representing low, medium, and high treatments): gray box represents data between the 25th and 75th percentiles, vertical capped lines (i.e., whiskers) represent data between the 5th and 95th percentiles, black dots represent data outside of the 5th and 95th percentiles, solid line in gray box indicates the median (50th percentile), and dashed line indicates the mean.

Analysis based on dispenser number was conducted because it corresponds with the simplified method of developing programs where set numbers are used per store. The number of dispensers per store—low, medium, or high label rates—did not have a significant impact on the average number of moths captured under MD (Kruskal–Wallis analysis of variance on ranks: H = 1.315, d.f. = 3, *p* = 0.725) or the percentage reduction (Kruskal–Wallis analysis of variance on ranks: H = 2.479, d.f.= 3, *p* = 0.479). The averages were 5.5 ± 1.5, 4.4 ± 1.4, 4.4 ± 0.8, and 3.5 ± 1.9 moths/trap/month and 80.3 ± 6.9, 90.3 ± 3.2, 81.1 ± 7.0, and 91.6 ± 2.6% reductions for the stores with 36 single, 54 single, 72 single, and MESO dispensers, respectively. However, because the stores did vary in size, this resulted in a range of volumes treated per dispenser within a dispenser number treatment. To determine if this impacted conclusions, we converted the data to the volume of space treated by a single dispenser. There was no significant relationship between the number of moths under MD and the volume treated per dispenser (ANOVA: F = 0.437, d.f. = 1, 32, *p* = 0.513) (Figure 5). Within a store over time, individual trap locations would have spikes of moth capture activity from time to time or took longer for moth activity to decrease after implementing the program, but there was no apparent increase in this variation with the decrease in the number of dispensers (Figure 6).

### 3.5. Evaluation of Disruption Mechanism

**Figure 7:**
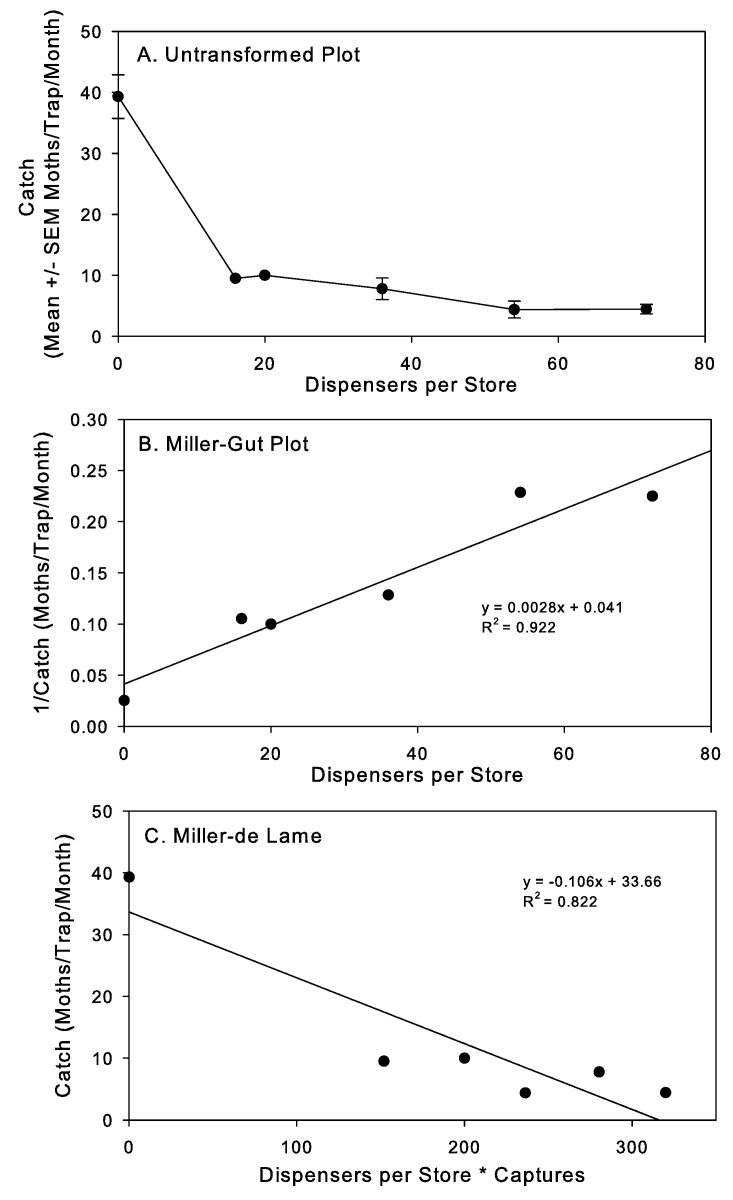
(**A**) Untransformed, (**B**) Miller–Gut, and (**C**) Miller–de Lame plots of the disruption profiles for *Plodia interpunctella* captures in pheromone-baited traps in retail pet stores under mating disruption (MD) treatments.

The plots of moth capture and dispenser number were consistent with relationships expected for competitive MD mechanisms as described in Miller et al. [26] using simulated data (Figure 7). The plot of the untransformed data (Figure 7A) was curvilinear, which resembles the profile for competitive attraction in Miller et al.’s study [26]. The initial steep drop in captures had leveled off by the lowest dispenser density tested, suggesting that even lower dispenser densities might still provide suppression. The Miller–Gut plot (Figure 7B) fit a linear function with a positive slope, which is consistent with competitive attraction mechanisms as opposed to a more concave pattern predicted with non-competitive mechanisms. The Miller–de Lame plot (Figure 7C) was also consistent with competitive attraction, since there was a good fit to the linear model, even with the large gap in data at low dispensers/store * moth capture values, and there was no evidence of a recurve in the data plot that would be expected for situations with non-competitive mechanisms [26]. Only monitoring data from periods of time when single dispensers were used in stores were included in this analysis, not the data using MESO dispensers, to facilitate the evaluation dispenser density on the ability of male moths to find traps.

Since the plots were consistent with a competitive mechanism, we proceeded to calculate dispenser activity (D_a_). Dispenser activity is the suppressive effect of a dispenser on the ability of males to find traps (i.e., the fraction of a store that a dispenser can theoretically reduce male captures by 50% from the level of capture in stores not under MD (C_max_)) [26]. From the Miller–Gut plot, this would be equal to 1/|x intercept|, which is D_a_ = 1/14.6 = 0.07. The absolute value of the slope of the Miller–de Lame plot also provides an estimate of D_a_, which, in this case, is 0.11. Thus, theoretically, one dispenser is capable of reducing moth captures by 50% in approximately 1/10th of a store (0.07–0.11). This leads to an estimate of dispenser application activity (D_Āa_) of 3.96, 5.94, and 7.92 for the 36, 54, and 72 dispenser rates per store, respectively. Converting this to the average size of a store in ha, to facilitate comparison with other field studies, this results in D_a_ = 0.02 and D_Āa_ equal to 4.22, 6.32, and 8.42 for the 36 (211 dispensers/ha), 54 (316 dispensers/ha), and 72 (421 dispensers/ha) dispenser rates per store, respectively.

## 4. Discussion

The MD treatments were effective at reducing *P. interpunctella* activity across the retail stores, and factors such as store chain, geographic distribution, initial moth density, and dispenser number did not significantly impact moth suppression. This suggests that a simplified and standardized MD program could be an effective management tactic for retail stores. The standardized approach to dispenser number and placement is an attractive option for corporate retail chains and pest management companies, since it could reduce the costs associated with the program and simplify the implementation and servicing of the program. However, the variation in the rate of reduction among the stores and spikes of pest activity under the MD programs suggest that there is room for improvement in pest suppression. A more customized MD program with enhanced supplemental activities such as sanitation and stock management could facilitate even better control. New MD application methods such as microencapsulated liquid sprays may also reduce labor associated with program implementation and provide ways to more effectively target treatments [25]. A standardized approach to MD implementation may be less likely to be successful in other types of facilities that are more variable in terms of volume, products, and physical layout. It is also possible that the variation in the results among stores could have resulted from genetic variation in the populations.

The introduction of the MD dispensers resulted in a large initial reduction in moth captures (67.8 ± 4.8%), and afterward, there was a more gradual reduction in captures over time. The reduction in moth captures under MD could have been caused by two factors: (1) fewer of the males that were present being able to find the traps (i.e., the treatment disrupting the ability to locate the pheromone source in the traps) and (2) fewer males being present (i.e., a reduction in the total population size). The initial drop observed in the first monitoring period after starting MD was most likely due to the impact of the MD dispensers on the ability of the males to find traps. This is because a one-month monitoring period, given *P. interpunctella’s* generation times, is not predicted to be long enough to detect decreased oviposition. For example, on a diet of bran, almonds, pistachios, or walnuts, the average *P. interpunctella* development time ranged between 28 and 47.2 days at 25 °C [29]. Store temperatures were not measured in our study, but Roesli et al. [28] reported that retail pet stores typically maintained temperatures between 20 and 25 °C. Other field studies of MD in food facilities have reported variable initial reduction results, ranging from almost complete shutdown to no detectable impact on male captures in traps [3,4,5,21]. Interpretation of these field trials is complicated because of variations in conditions within and among studies. Support for the hypothesis that the initial reduction was due to impacts on males’ abilities to locate traps comes from an evaluation of MD in a dried bean storage warehouse [23]. By measuring male moth captures in traps and mating success using sentinel females and oviposition using diet cups, this study found that male captures in traps was reduced by more than 50% two weeks after starting MD, but the proportion of oviposition cups with eggs was similar over this same period. After females already mated before the start of MD were predicted to no longer survive, no oviposition was detected in cups, and male captures in traps was low for the rest of the 13-week monitoring period, while male captures and oviposition in the cups continued in an untreated control building.

How MD treatments impact *P. interpunctella* male behavioral interactions with pheromone traps is not well understood and needs further investigation. Burks and Kuenen [30], in a study conducted in a 1000 m^3^ room, found that MD (high label rate) did not decrease *P. interpunctella* male captures in traps across a range of pheromone load rates. However, this study was conducted in a relatively small area, and the impact of MD on male moths’ abilities to find traps is likely to be greater in larger and more spatially complex landscapes typical in commercial food facilities. Pheromone lures in traps release pheromones at a greater rate than an individual calling female, and males are more effective at finding pheromone lures than females, so continued captures of males in traps does not necessarily indicate that they are also finding females at a similar rate. For example, in small 70 m^3^ structures, the number of males captured in traps with a calling female *P. interpunctella* was less than the number captured in traps with a pheromone lure containing 10 mg of ZETA, a higher rate than typically found in pheromone lures [30].

In our study, moth captures in traps continued after the initial reduction under MD, and although there was considerable variation among stores and among locations within a store, the overall trend was for average moth captures to gradually decrease. This trend was likely due to the reduction in the overall moth population in the retail stores. Continued moth activity could have resulted from (1) mating continuing to occur in populations under MD or (2) immigration of mated females into the treated space through active dispersal or inbound infested product. Mating disruption field evaluations that have included methods of monitoring female moth activity (e.g., sweep netting, unbaited traps, oviposition cups, or water traps) have also indicated that mated females are present under MD, albeit at a reduced level [4,5,20,21].

Mating can still occur under MD treatments for various reasons, most likely including spatial or temporal gaps in high pheromone concentrations allowing males to orientate toward females or males and females encountering each other without long-range attraction due to random movement or use of alternative short-range cues. High-density conditions, either store-wide or localized, can increase the chance of mating. *Plodia interpunctella* uses a range of cues over short distances to facilitate courtship and successful copulation [31], and we have observed that successful mating can occur under MD conditions when males and females are confined together in small arenas (Campbell, unpublished data). Silhacek et al. [32] observed in small, closed warehouses with air circulation, where it was speculated pheromone plume formation would be limited, that if walking males exhibited wing-fluttering behavior—associated with the presence of pheromone [33]—and contacted females, this triggered copulations. Sower et al. [34] observed that male *P. interpunctella*, when previously exposed to MD, did not orient to calling females even when 1 to 2 cm away, but copulation behavior was not inhibited if males directly contacted a female.

Conditions can exist inside food facilities that will produce localized high densities and an increased chance of encounters without long-range attraction. *Plodia interpunctella* activity and infestations can have a spatially patchy distribution [35,36], and infestations can occur inside packages and in cracks and crevices and other areas in the building structure where food material accumulates. This can create localized conditions with high densities and a higher probability that random encounters enabled by short-range attraction using visual, auditory, or non-ZETA-pheromone-based chemical cues could be used by males and females. However, some of these high-density impacts might be mitigated, since the last-instar larvae have a wandering phase before pupation, and females tend to move to vertical surfaces after adult emergence and before calling [32], which should reduce proximity between males and females. However, preferred substrates for pupation could also generate patchy distributions in emerging adults. For example, pupating *P. interpunctella* can often be found in pegboard used in retail displays and shelving and in corrugated cardboard. All of these factors could contribute to allowing a low population level to persist under MD treatments, but this also highlights how careful inspection and supplemental pest management tactics could enhance MD effectiveness.

Immigration of mated females into food facilities is another potential mechanism for the continued presence of pest populations under MD treatments and can occur by way of two primary methods: (1) active immigration via dispersal behavior and (2) passive immigration via human movement of infested materials. Active immigration can be important in some food facilities that have high levels of moth flight activity occurring outdoors, allowing these individuals to potentially make their way into structures and contribute to product infestations [37,38]. The retail stores in this study did not appear to be strongly impacted by active immigration from the surrounding landscape, given the lack of seasonality in moth captures observed (i.e., increased captures during warmer months).

Seasonal patterns in captures can result from fluctuations in outside populations producing resulting fluctuations in immigration rates. Other factors influencing trap capture variation could be the temperature inside the facility following seasonal patterns or moth diapause [37]. In facilities with seasonal patterns in moth activity, MD can still be effective in reducing the size of seasonal peaks if implemented prior to the start of seasonal moth activity [22]. Passive immigration of stored-product insect pests is a major factor in many parts of the food industry. Food products can become infested at different points in distribution channels, starting at the point of manufacture and continuing to the retail store, and *P. interpunctella* is highly effective at infesting packaged foods [39,40]. Shared distribution channels before arrival at the store could make the retail chain and location critical prediction factors if inbound infested material was a major contributor to population levels. These variables did not show significance in our analysis. However, given that infested inbound packages are expected to occur at a low percentage and to be stochastic, any impacts may be difficult to detect at our level of analysis. Another factor is that consumers return infested products to the retail stores, whether they become infested before or after purchase, and these are stored in receiving rooms before being returned to the manufacturer, creating another route for passive immigration. Retail stores are challenging environments for MD given these potential routes for continual moth immigration, despite this fact we still found a high level of MD efficacy. How the retail store manages these potential sources as part of a broader pest management program is likely to influence MD effectiveness, with tactics such as inbound product inspection, quick removal of infested packages, sanitation, and keeping doors closed and sealed contributing to reducing the successful immigration of mated females.

Mating disruption by competitive attraction, where males’ abilities to follow pheromone plumes are disrupted due to competing pheromone from MD dispensers, is the most common mechanism documented in the literature [27]. This determination is made using graphical plots derived on enzyme–substrate kinetic data developed by Miller et al. [26] and applied to field applications [27] and large-cage studies [41]. Here, we applied these graphical approaches to our data on the MD of *P. interpunctella* in retail stores and found that the patterns were consistent with competitive mechanisms being the predominant mode of action. This is the first report demonstrating the potential mechanism for *P. interpunctella* MD in field applications. Unlike previous experimental data used for this type of analysis [27,41], our zero-dispenser-treatment data was obtained during the pretreatment period rather than through controls that were run concurrently. We think that this approach is justified here, as with the overall experiment, because of the large amount of replication and the inherent high level of variability in moth captures across the stores and over time within the stores, but further evaluation confirming the predicted mode of action is needed. In addition, when evaluating MD mechanisms, it is important to note that MD is a complex process with multiple interrelated mechanisms that are impacted by a range of endogenous and exogenous factors [2,26]. Competitive disruption can result from several specific mechanisms, such as competitive attraction (false trail following), induced allopatry (males drawn away from females), and induced arrestment (reduced searching) [2]. A key point with competitive mechanisms of MD is that males can respond to females and traps under these treatments, which is consistent with data collected on *P. interpunctella*. Understanding MD mechanisms will help with further developing post-harvest MD programs and supports the call made by Miller and Gut [2] for progress in differentiating among MD mechanisms and the identification of critical causal actions of MD products.

Prior research measuring stored-product moth behavior under MD is limited and challenging to interpret concerning identifying MD mechanisms. Wijayaratne and Burks [42] found that even short exposures of male *P. interpunctella* to MD early in the scotophase resulted in an extended suppression in mating, which is more consistent with non-competitive disruption, but Burks and Kuenen [30] found that for male *P. interpunctella* that had interacted with MD dispensers, the response to female-baited traps was quickly restored after the removal of dispensers. Ryne et al. [17] found that male EAG antennal responses to pheromones decreased and became more variable under exposure to high-pheromone treatment. Time spent under MD treatment also appeared to impact the ability of male moths to locate pheromone lures and calling females [30]. Competitive disruption can also increase the time it takes for a male to successfully locate a female, which can impact longevity and oviposition [43]. Simulation of this effect has been demonstrated to have major impacts on stored-product insect progeny production and population growth [44,45,46], but effect in MD applications is not well understood. A less well studied area is the impact of MD treatment on female behavior, with female pheromone autorecognition potentially increasing or decreasing the effects of MD [47,48]. Gerken et al. [48] found strain differences in female *P. interpunctella* responses, with one strain decreasing calling behavior and the other increasing calling under MD, while both strains became less mobile. Shani and Clearwater [19] found that female *C. cautella* could be selected for increased pheromone production when exposed to MD conditions. More research is needed delving into the specific moth behavioral responses to each other, traps, and dispensers and the potential for interactions of different mechanisms to occur under MD treatments.

Under competitive mechanisms of MD, efficacy is predicted to be insect-density-dependent, with MD being less effective at high pest densities [1,49]. However, we found that the percentage reduction in *P. interpunctella* captures under MD was not significantly correlated with initial moth abundance. However, there was a suggestion in the data that there was a lesser reduction in captures at the highest initial densities. We also found that the percentage reduction did not change with density, except at the very lowest observed moth densities. There have been other experimental studies of *P. interpunctella* MD that have looked at moth density as a factor that support density dependence. Ryne et al. [17] found no difference in mating frequencies of *P. interpunctella* under MD between 0.6 moths/m^3^ and 1.9 moths/m^3^ (equal number males and females), although at both densities, mating frequency was lower than in the controls. In contrast, Sower et al. [34] and Sower and Whitmer [11] showed across a range of moth densities in small containers or rooms that the percentage of mated female *P. interpunctella* increased with increasing population size under MD conditions.

With *C. cautella*, another pyralid stored-product pest moth that responds to ZETA, after exposure to MD in small chambers, there were fewer mated females at the two lowest moth densities (3.0 moths/m^3^ and 5.9 moths/m^3^) than at the highest density (8.9 moths/m^3^) [50]. Variation in factors among these studies makes further analysis difficult, but the results do suggest that there is a moth density effect on MD effectiveness when relatively high moth densities are used and under more controlled and smaller-scale conditions. The lack of a relationship between moth density and MD efficacy observed in our study could have resulted from the range of densities in stores not being sufficiently broad enough to detect a pattern or from the fact that other variables introduced variation that obscured the relationship. Accurate determination of pest density is also challenging using pheromone-baited traps, since trends in captures are not necessarily linearly related to population density. From an applied perspective, our results suggest that MD should be effective in a relatively density-independent manner under moth population densities typically found in retail pet stores, but further evaluation will be needed for other types of facilities where higher moth densities can occur.

Dispenser density is another important variable in MD program application that we evaluated. Under a competitive mechanism of MD, the relationship between increasing pheromone concentration and efficacy is asymptotic [27]. Some of the implications of increasing dispenser number when competitive MD mechanisms are involved are discussed in Miller and Gut [2]. In our study in retail stores, we did not see any significant reduction in moth captures as the dispenser number increased from 36, to 54, to 72 dispensers per store. There was also not a significant relationship with volume treated per dispenser and number of moths captured. These results suggest that the tested dispenser densities were all on the higher end in terms of efficacy, where there are diminishing returns to increasing the number of dispensers. This could provide some cost savings, since a lower label rate could be used. Prior research has shown that pheromone concentration did impact mating success under controlled laboratory and small-room trials. Ryne et al. [17], in small-container trials, found a similar disruption of mating at both a low and high rate of MD spraying (doses of 0.75 and 3.75 mg/spray). Sower et al. [34] evaluated mating in an 89 m^3^ room and found that the *P. interpunctella* mating reduction relative to the control increased with increasing ZETA concentration. Hagstrum and Davis [12] found a reduction in the *C. cautella* mating rate with increasing pheromone release rate at a low-moth-density treatment but not at a high-moth-density treatment. Another factor is whether releasing the synthetic pheromone in relatively lesser amounts from a greater number of locations or relatively greater amounts from fewer locations will be more effective [26,40].

## 5. Conclusions

This was the largest replicated MD assessment for management of a post-harvest pest and provides valuable foundational and applied insights into the process. Our results show that an MD program can provide an acceptable level of pest suppression over time. The data generated provided us an opportunity to identify competitive disruption as the primary MD mechanism, which provides a valuable tool for guiding future research and application. MD takes time to be fully effective in retail stores, likely because of immigration of mated individuals and the spatial complexity of the facilities and patterns of moth distribution allowing for mating to still occur in stores under MD. We showed that MD was effective across the range of moth densities that occurred in these retail stores, which were, on average, considered to have a high level of moth activity, and we showed that the low-label-rate treatment could be just as effective as higher rates under these retail conditions. This could enable these programs to be implemented at a reduced cost. The evaluation of MD efficacy and factors that influence it on a commercial scale can realistically only be tackled by collaboration among researchers, manufacturers, retailers, and pest management professionals from different parts of the food industry.

## Figures and Tables

**Table 1:** Store Locations (store numbers in bold are chain #1 and those not bold are chain #2). * indicate that rate was lower than the recommended range on the label (1 dispenser per 100 to 300 m^3^ for single dispensers and 1 meso dispenser per 400 to 1200 m^3^ for the MESO). The US climatic regions were Central (Ohio Valley) (C), Southeast (SE), Southwest (SW), Northeast (NE), and South (S). In phase 1 dispensers were placed either on shelves or ceiling, but in all other phases there were placed on shelves.

Store	US Climatic Region	Volume (m^3^)	Pre-MD Period (Months)	Phase 1 Period (Months)	Phase 1 Dispenser Number (Location)	Phase 2 Period (Months)	Phase 2 Dispenser Number (Meso Number)	Phase 2 Application Rate (Volume (m^3^) per Meso)	Phase 3 Period (Months)	Phase 3 Dispenser Number (Meso Number)	Phase 3 Application Rate (Volume (m^3^) per Dispenser)
1	C	17,384	8	16	36 (shelf)	6	36 (9 meso)	1932 *	9	36	483 *
2	C	10,295	2	16	36 (shelf)	6	36 (9 meso)	1144	9	36	286
3	SE	14,100	4	na	na	5	36 (9 meso)	1567 *	10	36	392 *
4	SE	14,180	4	na	na	5	36 (9 meso)	1576 *	10	36	394 *
5	SE	10,362	4	na	na	5	36 (9 meso)	1150	10	36	288
6	SE	10,212	8	15	16 (ceiling)	7	36 (9 meso)	1135	9	54	189
7	SE	14,062	8	16	36 (ceiling)	6	36 (9 meso)	1562 *	9	54	260
8	SW	14,072	3	8	36 (ceiling)	6	36 (9 meso)	1564 *	9	54	261
9	SE	13,889	4	na	na	5	36 (9 meso)	1543 *	10	54	257
10	S	12,572	<1	na	na	na	na	na	9	64 (16 meso)	196
11	C	15,604	2	16	20 (shelf)	6	36 (9 meso)	1734 *	9	72	217
12	SW	10,300	3	8	36 (ceiling)	6	36 (9 meso)	1144	9	72	143
13	SE	10,153	4	na	na	5	36 (9 meso)	1128	10	72	141
14	SE	8637	4	na	na	5	36 (9 meso)	960	10	72	120
15	S	15,740	<1	na	na	na	Na	na	10	80 (20 mesos)	197
16	S	16,642	<1	na	na	na	Na	na	10	84 (21 mesos)	198
17	SE	6415	4	na	na	5	36 (9 meso)	713	10	36	178
18	SE	8495	4	na	na	5	36 (9 meso)	944	10	36	236
19	SW	9378	4	na	na	6	36 (9 meso)	1042	9	36	260
20	SW	7646	4	na	na	6	36 (9 meso)	850	9	36	212
21	NE	10,590	4	na	na	6	36 (9 meso)	1177	9	36	294
22	S	7929	<1	na	na	na	Na	na	10	40 (20 doubles)	198
23	S	8325	<1	na	na	na	Na	na	10	42 (21 doubles)	198
24	S	8722	<1	na	na	na	Na	na	10	44 (22 doubles)	198
25	SE	8628	4	na	na	5	36 (9 meso)	959	10	54	160
26	SE	13,427	4	na	na	5	36 (9 meso)	1492 *	10	54	249
27	NE	8495	4	na	na	6	36 (9 meso)	844	9	54	157
28	SE	7815	4	na	na	6	36 (9 meso)	868	9	54	145
29	SE	8877	4	na	na	5	36 (9 meso)	896	10	72	123
30	SW	8509	4	na	na	6	36 (9 meso)	945	9	72	118
31	SW	8530	4	na	na	6	36 (9 meso)	948	9	72	118
32	NE	7702	4	na	na	6	36 (9 meso)	856	9	72	107
33	SE	9854	4	na	na	6	36 (9 meso)	1095	9	72	137

## Data Availability

The original data presented in the study are openly available on Ag Data Commons, USDA ARS National Agricultural Library, at https://doi.org/10.15482/USDA.ADC/28887146.
